# AI Integration in Undergraduate Medical Education: Qualitative Study of Faculty Perspectives in the United Arab Emirates

**DOI:** 10.2196/90192

**Published:** 2026-08-13

**Authors:** Azhar T Rahma, Uffaira Hafeez, Munawar Farooq

**Affiliations:** 1 Institute of Public Health, College of Medicine and Health Sciences, United Arab Emirates University (UAEU), Al Ain, United Arab Emirates; 2 Department of Internal Medicine, Emergency Medicine Section, College of Medicine and Health Sciences, United Arab Emirates University (UAEU), Sheik Khalifa Bin Zayed St - 'Asharij - Shiebat Al Oud - Abu DhabiAl Ain, Abu Dhabi, 0000, United Arab Emirates, 971 50 779 1906

**Keywords:** artificial intelligence, medical education, medical curriculum, medical school, AI ethics, medical faculty, United Arab Emirates, UAE

## Abstract

**Background:**

AI is transforming health care, creating an imperative to integrate AI into medical education. While student perspectives are well-studied, faculty views, particularly in non-Western contexts, remain underexplored.

**Objective:**

This qualitative study explores the perspectives of 10 medical faculty members from 2 institutions in the United Arab Emirates on integrating AI into undergraduate medical education.

**Methods:**

This multi-institutional qualitative study used purposive and reflexive sampling to recruit faculty from both public and private medical universities in the United Arab Emirates. Semistructured interviews were conducted with 10 faculty members involved in curriculum design, teaching, or assessment. Data collection followed COREQ (Consolidated Criteria for Reporting Qualitative Research) guidelines. Data were analyzed using a mixed inductive-deductive approach guided by the thematic analysis framework of Braun and Clarke. Findings were interpreted using the FACETS (Form, AI Use Case, Context, Education, Technology, and SAMR: Substitution, Augmentation, Modification, Redefinition) framework, which supported a structured examination of AI integration across different dimensions of teaching and learning.

**Results:**

Faculty primarily used generative AI tools, such as ChatGPT, for content creation, assessment development, and teaching support, reflecting a preference for accessible and general-purpose technologies. AI was mainly used to enhance teaching efficiency and support student learning, including personalized study planning and practice activities. Its application extended across preclinical and clinical contexts, with strong emphasis on adapting content to local cultural and ethical norms. While AI was perceived to improve efficiency and alignment between teaching and assessment, concerns were raised regarding equity, overreliance, and variability in student use. Overall, adoption remained focused on enhancing existing practices, with limited transformative use but recognition of future potential for more advanced applications.

**Conclusions:**

The UAE medical faculty demonstrate cautious optimism toward AI integration, recognizing its potential to enhance educational efficiency and personalization while emphasizing the critical importance of cultural contextualization. Current implementation remains at early adoption stages, focused on enhancement rather than transformation. Successful integration requires faculty development, context-sensitive policies, and equitable implementation strategies that address both technological and sociocultural dimensions of AI adoption in medical education.

## Introduction

AI refers to computer systems capable of performing tasks traditionally requiring human intelligence, such as learning, reasoning, and decision-making [1]. In health care, AI is increasingly influencing clinical practice by enhancing diagnostic accuracy, clinical decision support, and predictive analytics [2,3]. Significant advancements have been demonstrated across multiple specialties [4-8], prompting discussions about how AI may reshape, supplement, or shift professional medical roles [9]. Globally, governments and health systems, including in the United Arab Emirates, are investing in AI-driven innovations and workforce readiness initiatives [10,11]. As health care becomes increasingly digitalized, future physicians will be expected to collaborate effectively with AI systems, critically evaluate their reliability, and make informed clinical judgments in augmented practice environments [12]. This has led to calls for integrating AI-related knowledge, critical appraisal skills, and ethical reasoning into undergraduate medical education [13].

Several studies have explored AI’s potential to enhance medical teaching across diverse disciplines. For example, augmented reality has been used to improve the understanding of complex anatomical structures [14]; AI supports diagnosis and monitoring in ophthalmology [15] and lesion recognition in dermatology [16], provides adaptive simulation-based training in surgery [17], and enhances image-based interpretation in radiography through deep learning [18]. More recently, research has focused on generative AI and large language models (LLMs) in medical education. These studies describe applications such as supporting simulation-based training, improving students’ digital literacy and ethical competencies [19], answering medical licensing exam questions [20], and comparing AI-generated versus expert-generated questions [21]. Beyond assessment, generative AI has been explored as a virtual mentor and educational support tool [22], delivering personalized formative feedback efficiently [23], enhancing case-based learning, and aligning AI-based grading with teacher evaluations [24]. LLMs have also demonstrated potential in enabling authentic assessments by providing immersive, real-world evaluation experiences [25].

Integrating AI into medical education represents a significant pedagogical shift, with students as the primary recipients of innovation and faculty as the key drivers of its implementation. Evidence suggests that students generally hold positive attitudes toward AI, recognize its potential to enhance learning and diagnostic reasoning, and advocate for structured training within the curriculum [26-28]. However, faculties face substantial challenges at both personal and institutional levels [29]. Multicenter studies indicate that, despite generally positive faculty attitudes, significant gaps remain in formal AI training and knowledge [30]. Faculty engagement is critical for successful curricular reform, as their beliefs, readiness, and professional development directly influence adoption and sustainability [31]. Therefore, efforts to integrate AI must address faculty concerns and actively involve them in the process. Moreover, effective adoption requires a contextualized approach that considers local educational, cultural, and institutional factors [32]. Despite the growing body of literature on AI in medical education, most studies have focused on technological applications, expert perspectives, or student perspectives, with comparatively limited attention to faculty experiences, particularly in the Middle East and the UAE context. There is a lack of context-specific evidence on how faculties perceive their readiness, roles, and challenges in integrating AI into existing curricula. Therefore, this study aims to explore medical faculty perspectives on the integration of AI into undergraduate medical education in the United Arab Emirates, focusing on their experiences, perceived readiness, and implementation challenges. The study applies the FACETS (Form, AI Use Case, Context, Education, Technology, and SAMR: Substitution, Augmentation, Modification, Redefinition) framework to move beyond descriptive accounts and provide a theoretically informed, multidimensional analysis of AI integration in medical education.

## Methods

### Study Design

A qualitative descriptive approach was used to explore faculty perspectives on integrating AI into medical education in the United Arab Emirates. This study followed the COREQ (Consolidated Criteria for Reporting Qualitative Research) guidelines [33]. Semistructured interviews were conducted to capture faculty experiences, beliefs, and concerns regarding AI integration while allowing flexibility to pursue emerging topics of significance to participants.

### Epistemological Position

This study adopts a critical realist epistemological stance, acknowledging that while participants’ experiences and perspectives represent real phenomena, our interpretation and presentation of these experiences are inevitably shaped by our theoretical lenses, disciplinary backgrounds, and the analytical frameworks we employ.

### Sampling

A purposive sampling approach was employed to identify and recruit faculty members from the medical schools in the United Arab Emirates: either a government-funded public university or a private, internationally affiliated institution, located in the United Arab Emirates. Eligible participants were those actively involved in curriculum development, teaching, or assessment. Invitations were distributed via departmental mailing lists and targeted emails to faculty with relevant roles.

**Figure 1.**
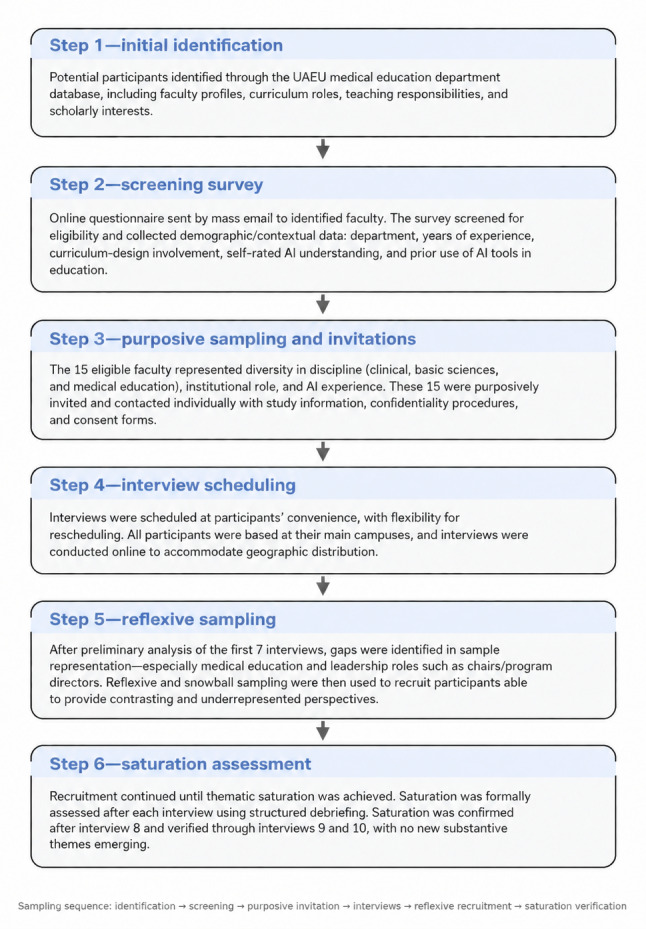
Participant recruitment flow: qualitative study of United Arab Emirates University faculty.

An initial pool of 47 faculty members was identified, of whom 22 responded to a screening questionnaire assessing academic roles, experiences, and familiarity with AI. Fifteen met the inclusion criteria, and 7 participated in the initial round of interviews. Following preliminary analysis, additional participants were recruited using reflexive and snowball sampling to ensure representation across disciplines (clinical, basic sciences, and medical education) and varying levels of AI experience. A total of 10 faculty members were interviewed. The term “key informants” refers to participants selected based on their roles, experiences, or departmental affiliations relevant to the study (Figure 1).

Recruitment continued until thematic saturation was reached. Saturation was observed after 8 interviews and confirmed with 2 additional interviews, with no new themes emerging.

All interviews were conducted online. Participants were based at their respective institutions, and the inclusion of faculty from both public and private settings allowed for a range of perspectives.

### Data Collection

This study took place in the United Arab Emirates between January and April 2025. We recruited participants from institutions that offer MBBS programs accredited by the Commission for Academic Accreditation.

An initial online questionnaire was used to screen participants and collect demographic and contextual information. It served both as a screening tool to identify eligible participants based on curriculum involvement and AI knowledge and as a means of gathering background data, including departmental affiliation, years of experience, curriculum involvement, self-rated AI understanding, and prior experience with AI tools in education.

Faculty who met the inclusion criteria and expressed interest were invited to participate in the interviews.

Data were collected through semistructured online interviews using a predefined interview guide (Multimedia Appendix 1), which was reviewed by experts in medical education and qualitative research. Verbal informed consent was obtained before each interview. Interviews lasted between 30 and 60 minutes and were audio-recorded and transcribed verbatim. Audio recordings were deleted after transcription to ensure confidentiality.

Interviews were conducted by AR, a public health faculty member with experience in qualitative research, with support from MF and UH. Some participants were known to the research team in a professional context. However, participation was voluntary and interviews were conducted independently of prior relationships. Participants were informed about the study purpose, confidentiality measures, and their right to withdraw.

Field notes were recorded during and after interviews to capture contextual observations and initial reflections. Transcripts were retained to support reflexivity, allowing repeated engagement with the data, reflection on researcher assumptions, and maintenance of an audit trail of analytical decisions. Transcripts were returned to participants for verification. NVivo (version 15; Lumivero) was used to support data management and analysis.

### Data Analysis

Data were analyzed using reflexive thematic analysis, informed by the 6-phase approach of Braun and Clarke [34]. After transcription, the transcripts were read repeatedly to ensure familiarization with the data. Coding was conducted inductively, with codes generated directly from participants’ accounts rather than from a preexisting framework.

The initial coding was carried out independently by 2 analysts (AR and UH). The analysts met regularly to compare coding decisions, discuss interpretations, and refine the developing coding structure through an iterative process of discussion and review.

Codes were then grouped into candidate themes by identifying patterns of shared meaning across the dataset. These themes were reviewed against the coded extracts and full transcripts to ensure coherence, distinctiveness, and relevance to the research question. Themes were then defined, named, and organized into a coherent analytic narrative.

Once initial themes were developed, the FACETS framework was used as an interpretive lens to further structure and refine the findings [35,36]. This allowed the analysis to remain grounded in participants’ accounts while also supporting a more structured interpretation of AI integration across multiple dimensions.

### Researcher Reflexivity and Positionality

Prior to the study, the research team engaged in reflexivity discussions to consider how their disciplinary backgrounds, professional roles, and assumptions might influence the research process. The team included a public health faculty member with qualitative research experience (AR), clinical faculty members involved in medical education and curriculum-related work (MF and UH), and a medical education scholar (SW). These backgrounds provided complementary perspectives related to curriculum development, educational innovation, and the integration of AI in health professions education.

The team acknowledged a shared preconception that AI holds important potential for medical education, while also recognizing concerns related to ethics, equity, overreliance, and contextual appropriateness within the UAE setting. We also recognized that our academic positions and prior engagement with medical education may have shaped the issues we considered important and the meanings we foregrounded during the analysis. To mitigate the influence of these assumptions, we used a semistructured interview guide, maintained reflexive memos throughout data collection and analysis, and engaged in regular analytic discussions to ensure that findings remained grounded in participants’ accounts.

### Trustworthiness

Several strategies were used to enhance the trustworthiness of the study. Credibility was supported through in-depth, semistructured interviews, prolonged engagement with the transcripts, iterative analytic discussions among the research team, and the inclusion of verbatim quotations to illustrate the themes. Dependability was strengthened through a transparent and documented analytic process, including coding records, reflexive memos, and an audit trail that tracked the development of themes from raw data to final interpretation. Confirmability was addressed through reflexive practice, explicit acknowledgment of researcher assumptions, and grounding interpretations in participants’ own words, as well as returning the transcript for some participants for validation. Transferability was supported by providing a clear description of the study context, participant characteristics, institutional settings, sampling strategy, and recruitment process, allowing readers to assess the relevance of the findings to similar contexts.

### Ethical Considerations

The study was approved by the UAE University Ethics Board (reference ERSC_2024_4790). Participation was voluntary, and all participants provided informed consent. No financial or other compensation was provided to participants. Data confidentiality and anonymity were strictly maintained throughout the research process.

## Results

### Perspectives on Integration and Current Practices

#### Overview

The study included 10 faculty members and represented a range of disciplines, including emergency medicine, internal medicine, public health, obstetrics and gynecology, pathology, and medical education. Most had extensive involvement in curriculum design, with a few serving in a curriculum leadership capacity. The self-rated understanding of AI ranged from “moderate” to “good” (Table 1).

**Table 1.** Characteristics of key informants (N=10).

Participant ID	Department	Role in curriculum design	University type	Self-rated understanding of AI
KI-01	Emergency medicine	Extensive	Public	Good
KI-02	Internal medicine	Limited	Public	Moderate
KI-03	Public health	Extensive	Public	Moderate
KI-04	Medical education	Extensive	Public	Moderate
KI-05	Internal medicine	Extensive	Public	Expert
KI-06	Obstetrics and gynecology	Extensive	Public	Good
KI-07	Pathology	Lead	Private	Moderate
KI-08	Medical education	Moderate	Public	Good
KI-09	Medical education	Extensive	Public	Good
KI-10	Medical education	Moderate	Public	Good

**Figure 2.**
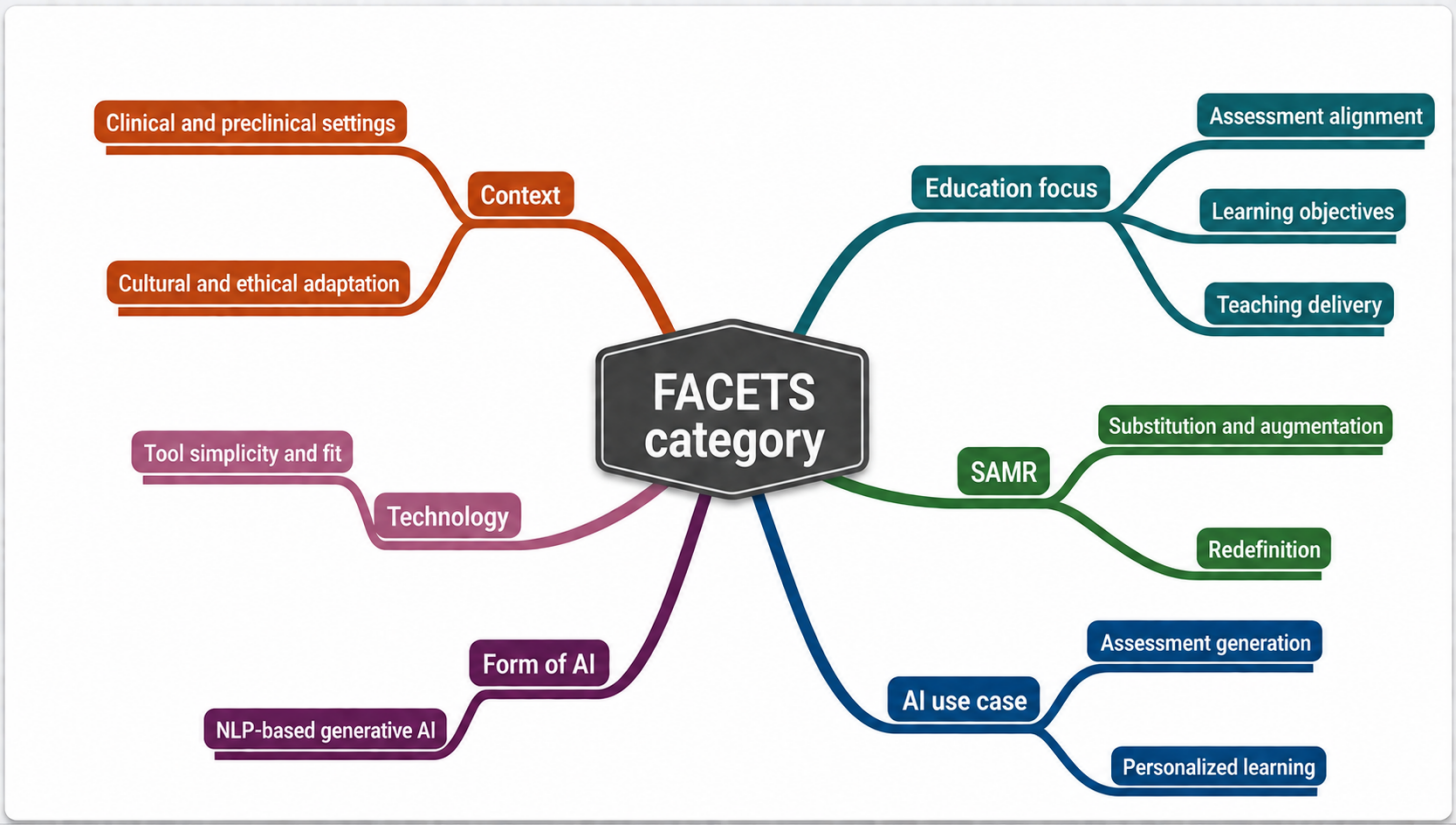
Faculty perspectives on themes related to strategically integrating AI in UAE medical education. FACETS: Form, AI Use Case, Context, Education, Technology, and SAMR; NLP: natural language processing; SAMR: Substitution, Augmentation, Modification, Redefinition.

The following findings are organized according to the FACETS framework (Figure 2). Beyond identifying where and how AI was being used, the analysis also revealed how faculty interpreted the meaning, limits, and implications of this integration within their specific institutional and sociocultural context.

This figure presents the 6 themes identified through the reflexive thematic analysis and shows how they were organized using the FACETS framework and interpreted alongside the SAMR model. The FACETS domains are Form (type of AI used), AI use case (how AI was applied), Context (where and under what sociocultural conditions AI was used), Education (teaching, learning, and assessment functions), Technology (tools and platforms adopted), and SAMR (level of educational technology integration). The figure shows that faculty perspectives clustered around accessible generative AI tools, especially natural language processing (NLP) applications, with reported uses in teaching support, assessment, and personalized learning. Participants emphasized the importance of contextualization to the UAE setting, including cultural, ethical, and language considerations. Across the dataset, the current AI use was interpreted mainly at the substitution and augmentation levels, with more limited examples of Modification and anticipated future potential for Redefinition.

#### (F)—Form of AI: A Monoculture of Generative NLP Tools

The analysis revealed a distinct homogeneity in the form of AI being adopted, which is almost exclusively centered on NLP-based generative AI, with ChatGPT as the dominant platform. This indicates that faculty are gravitating toward accessible, general-purpose tools rather than specialized educational software. The primary use of these tools was to act as an efficiency engine for content creation and simplification. Faculty leveraged them not for complex analytical tasks but to rapidly generate and refine textual and conceptual materials, effectively using AI as a powerful, on-demand assistant to streamline their workflow.

*Even using the free version... you can engineer a prompt for a lecture or concept and ask it to explain it in your own language and level.*

[KI-07]

*AI like ChatGPT is my assistant when writing problem-based cases*.

[KI-09]

#### (AI)—AI Use Case: Augmenting Educator Productivity and Enabling Student Personalization

The use cases for AI crystallized into 2 dominant patterns: enhancing faculty productivity and facilitating student-centered learning. For educators, the most significant application was in assessment generation and content authoring. Faculty reported using AI as a collaborative draftsperson to create a wide range of assessment items, from multiple-choice questions (MCQs) to clinical scenarios. This use was primarily motivated by a need for efficiency and diversity, allowing them to quickly generate a larger and more varied pool of teaching materials while ensuring alignment with learning objectives.

*I use ChatGPT to generate MCQs and essay questions across Bloom’s taxonomy*.

[KI-07]

*We’ve used it to create questions that match learning outcomes in less time*.

[KI-10]

Conversely, faculty members observed that students were leveraging the same tools for personalized learning. Students proactively used AI to create custom study plans, deconstruct complex topics, and generate adaptive practice questions, effectively creating a personal tutor. This divergence in use cases highlights AI’s dual role: as a productivity tool for instructors and an adaptive learning scaffold for students.

*It gives different versions every time you prompt it. That’s powerful for revising topics*.

[KI-10]

*Students ask AI for feedback or use it to rehearse for OSCEs.*

[KI-04]

#### (C)—Context: Pervasive Application Demands Conscious Localization

The context of AI application was notably broad, spanning both preclinical and clinical phases of the curriculum. Faculty reported using AI to develop materials for problem-based learning and team-based learning, indicating its utility across diverse pedagogical frameworks. This demonstrates that AI is not confined to a single teaching methodology but is seen as a flexible resource adaptable to various educational settings.

*We are already using AI in developing simulation-based clinical cases for team-based learning.*

[KI-07]

*AI offers real support for students preparing for bedside clinical reasoning*.

[KI-04]

More significantly, a powerful and recurring subtheme was imperative for culturally and ethically relevant integration. Participants universally emphasized that the default outputs of global AI models were insufficient for their context. They stressed the necessity of actively localizing content to align with the UAE’s specific socioreligious values, particularly concerning family structures, language, and ethical norms. This was not seen as a minor adjustment but as a fundamental requirement for the responsible and effective use of AI in their educational environment.

*You cannot just rely on global AI content. You must contextualize for family, religion, and language*.

[KI-06]

*Localization is not optional, it is essential for meaningful learning in our setting*.

[KI-05]

Participants also provided some examples of what cultural and ethical localization might look like in practice. One participant linked localization to personalized learning systems that could monitor learner progress and identify strengths and weaknesses, while also noting the privacy implications of such systems:

*Learning and having eventually soon having systems that help with tracking learners, tracking their progress. Personalized learning… it can be much easier to identify students who are progressing faster than others and what areas exactly are their weaknesses and strengths.*

[KI-09]

The same participant raised an important ethical concern.

*There is one huge negative there, or not negative but concern, which is that the learners have to give up some privacy… have to agree that this system is going to monitor you.*

*Let’s say we want more restrictions, especially ethical, like an Islamic kind of thing… maybe we want to add more sections to the engine. This should be feasible, and then we want to feed it with our own… institution, feed it with our own data and make use of it definitely.*

[KI-09]

These examples suggest that localization was discussed not only as a general principle but also as an emerging practical consideration, even if not yet implemented as a formalized institutional process.

#### (E)—Education Focus: Streamlining Delivery and Reimagining Assessment

The educational focus of AI use manifested in 2 key areas: refining assessment strategies and enhancing curriculum delivery. In the realm of assessment, faculty reported that AI was not just a tool for creating more questions, but for creating better-aligned and more authentic evaluations. By automating the generation of standard items (like MCQs), AI was perceived to free up faculty time and cognitive resources to focus on designing complex assessments that measure higher-order thinking skills, such as reflection and clinical problem-solving.

*We can finally return to authentic assessments—reflection, problem-solving, not just MCQs*.

[KI-08]

*AI assists me in validating that my questions match course objectives.*

[KI-01]

In terms of teaching and curriculum delivery, AI was integrated as a core workflow tool for instructional design. Faculty used it to draft lesson outlines, generate ideas for learning activities, and even simulate dialogue for feedback conversations. This use case positions AI as a collaborative thought partner in the planning stages of teaching, helping to structure and enrich the educational experience before it even reaches the classroom.

*AI is part of my teaching workflow now. It drafts outlines and gives me ideas for activities*.

[KI-02]

*I even use it to match learning goals with appropriate teaching methods*.

[KI-01]

Analytically, this shows that faculty are leveraging AI to enhance both ends of the educational process, such as the initial design of instruction and the final evaluation of learning, thereby strengthening the overall alignment of the curriculum. Analytically, this suggests that faculty are using AI not only to reduce workload but also to reinforce constructive alignment between teaching, assessment, and learning outcomes. In this sense, AI is being positioned as a tool that may support pedagogical coherence, even where its use remains primarily supportive rather than transformative.

#### (T)—Technology: A Pragmatic Approach Favoring Accessibility Over Specialization

The technology dimension revealed a distinctly pragmatic and cautious approach to tool selection. Rather than enthusiastically adopting a wide array of specialized educational AI platforms, faculty exhibited a strong preference for a few accessible, general-purpose tools such as ChatGPT, Notion AI, and Copilot. This suggests that low barriers to entry, including cost, ease of use, and immediate utility, are more significant drivers of adoption than advanced, niche features.

A notable finding was the explicit caution against technological overload. Faculty voiced a strategic reluctance to invest time in learning numerous platforms, advocating instead for a focused and mastery-based approach to a select few tools that demonstrably improved their core tasks.

*We should not get lost in fancy tools. Just choose what improves your teaching or assessment.*

[KI-01]

*Using AI has to be realistic—we can’t expect all tools to work with our curriculum*.

[KI-09]

This pragmatic stance suggests that faculty adoption is shaped not by enthusiasm for technological novelty alone but by selective judgment about feasibility, usefulness, and curricular fit. It also indicates that institutional AI strategies may be more effective when they emphasize a focused, sustainable set of tools rather than a broad and potentially overwhelming range of platforms. This apparent tension reflected a distinction between present feasibility and future possibility. Faculty favored simple tools for current use because they were practical and manageable, while viewing more transformative AI applications as contingent on future training, infrastructure, and institutional support.

#### (S)—SAMR Model of Integration

Analyzing the data through the SAMR model revealed that current AI integration is predominantly at the substitution and augmentation levels, indicating a focus on enhancing efficiency rather than achieving pedagogical transformation. At the substitution level, AI directly replaced existing tools, for instance, in drafting assessment questions.

*We used to spend hours on question writing—now AI does the first draft.*

[KI-08]

At the augmentation level, AI provided functional improvements to existing tasks, such as generating more diverse question formats or providing faster feedback, thus augmenting faculty capabilities.

*It augments how we teach—faster feedback, more questions, better materials*.

[KI-08]

While instances of modification (significantly redesigning tasks) were rare, faculty optimism pointed toward the potential for redefinition (creating previously inconceivable tasks). They envisioned AI-powered simulations for clinical communication or adaptive tutoring systems, suggesting a trajectory toward more transformative integration in the future, even if not yet realized in practice.

*I see potential in AI chat simulations for clinical communication training*.

[KI-03]

*Soon, AI will co-facilitate reflective learning and structured feedback.*

[KI-07]

This analysis, through the SAMR lens, confirms that while AI is a valuable supportive tool, its use has not yet fundamentally redefined educational paradigms in this context. Analytically, this pattern indicates that current AI adoption remains concentrated at the level of workflow enhancement rather than pedagogical redesign. The significance of this finding is that faculty are beginning to integrate AI into routine educational practice, but most uses have not yet altered the underlying structure of teaching and assessment in a transformative way.

### Strategic Priorities for Future Implementation

For AI to be a constructive force in medical education, participants proposed that 2 interlinked priorities must be addressed: faculty preparedness and the prevention of inequities.

#### Faculty Readiness and Development

Faculty members call for structured training programs on prompt engineering, ethical use, and curriculum alignment.

*Faculty development is urgently needed; many still don’t know how to prompt properly.*

[KI-02]

*We need structured workshops on how to use AI ethically and effectively.*

[KI-04]

#### Equity and Overdependence Risks

Equally important is the implementation of strategies to mitigate the risks of digital disparities and academic misconduct. Faculty expressed concerns about performance gaps resulting from digital disparities and unethical shortcuts taken by students.

*AI may widen the performance gap between high- and low-GPA students.*

[KI-04]

*Some are copying AI responses without understanding; it’s a dangerous trend.*

[KI-09]

Together, these priorities show that faculty do not view AI integration as a purely technical implementation challenge. Rather, they frame it as an institutional and pedagogical transition requiring capability building, ethical safeguards, and attention to unequal patterns of access and use. This helps explain why participants’ views were simultaneously optimistic about AI’s potential and cautious about its educational consequences.

## Discussion

### Overview of AI Integration in UAE Medical Education

Faculty in this study generally reported positive perceptions of AI integration in medical education in the United Arab Emirates, particularly in relation to assessment, feedback, lesson planning, and personalized learning. At the same time, participants highlighted concerns regarding ethical considerations, regulatory oversight, and the need for culturally appropriate implementation. The findings suggest that AI integration in UAE medical education is expanding, with both faculty and students benefiting from its accessibility and versatility. The current use remains largely focused on NLP-based tools such as ChatGPT for tasks including content authoring, assessment generation, feedback, and communication simulation. Faculty also expressed optimism about the potential of AI-powered simulations and adaptive tools for communication and reflective learning. AI applications now span both preclinical and clinical modules, with notable innovations in team-based and problem-based learning environments. Although AI supports more personalized learning and better alignment between assessments and learning outcomes, participants raised concerns about equity and overreliance, emphasizing that unequal access, differences in digital literacy, and potential misuse could widen performance gaps among students.

### Alignment With International Literature

These findings are consistent with international literature on the expanding role of generative AI tools, particularly LLMs such as ChatGPT, in medical education. Similar to other studies, participants recognized AI’s potential to enhance productivity, deepen understanding of medical concepts, and enrich learning experiences for both educators and students [37]. Faculty primarily use AI to streamline routine tasks, such as creating MCQs and preparing lectures, rather than for full pedagogical transformation, which is consistent with existing literature [35]. While the international literature increasingly emphasizes the role of AI in enabling personalized learning through real-time feedback and adaptive content delivery [38-40], participants in this study expressed these possibilities more as future aspirations than as fully realized practices [38-40].

### Cultural Localization and Ethical Considerations

While much of the Western literature focuses on concerns about data privacy and academic integrity [41], the findings of this study suggest a relatively stronger emphasis on responsible and context-sensitive adoption. Faculty perspectives reflected a balance between recognizing AI’s educational potential and maintaining pedagogical integrity, indicating that adoption within the UAE context may be shaped not only by technological capability but also by institutional priorities and broader sociocultural considerations. This perspective supports calls for context-sensitive AI integration strategies, particularly in non-Western and culturally diverse settings [42,43]. Participants further expressed concerns regarding equity and the risk that AI could widen existing educational gaps, reflecting broader literature on the digital divide in AI usage [44]. Overreliance on AI tools emerged as another potential challenge, with participants noting the risk of undermining critical thinking and independent learning skills [45]. Finally, faculty emphasized a strong need for structured professional development, as the lack of formal training may hinder effective AI integration into teaching practice [46].

### Theoretical Implications

Viewed through the FACETS framework, this study highlights the multidimensional nature of AI adoption in medical education. Faculty recognized AI’s value in supporting diverse educational forms such as content generation and personalized learning and in enabling a range of practical AI use cases that align learning outcomes with curricular goals while maintaining ethically and technologically sound practices. In the UAE context, ethics and curricular contextualization were especially emphasized, reflecting the importance of culturally aligned, locally regulated, and pedagogically coherent implementation.

These findings extend the FACETS framework by demonstrating how contextual factors influence each dimension. In particular, cultural expectations, regulatory considerations, and institutional policies influence how AI tools are selected (technology), applied (AI use case), and embedded within teaching practices (education focus), highlighting that effective AI integration depends not only on technological readiness but also on alignment with institutional and sociocultural norms [47].

Within this framework, the SAMR model illustrates the progression of AI adoption. Early integration occurs primarily at the substitution and augmentation levels, where technology enhances efficiency without altering pedagogy [36]. Faculty described using AI to automate tasks such as question generation, feedback preparation, and lesson planning, emphasizing that AI currently functions as a supportive tool rather than a transformative one. At the augmentation level, AI provides functional enhancements such as adaptive explanations and improved assessment design that extend faculty capabilities without altering instructional design [36]. The predominance of augmentation-level use may be explained by several interrelated factors. First, limited formal training and a lack of structured faculty development may constrain educators’ ability to integrate AI in more pedagogically transformative ways. Second, institutional and regulatory uncertainties, including concerns about academic integrity, data privacy, and ethical use, may encourage cautious adoption. Third, infrastructural and curricular constraints, such as limited access to advanced AI tools or a lack of integration within existing curricula, may further restrict progression toward higher levels of the SAMR model. Together, these factors contribute to a pattern of incremental rather than transformative adoption.

Some faculty, however, expressed confidence in AI’s potential for more advanced use, reflecting early movement toward modification and redefinition. At the modification stage, they envisioned AI supporting more interactive, student-centered learning through adaptive case discussions or personalized tutoring. At the redefinition stage, they anticipated AI-powered simulations and tailored feedback, enabling entirely new learning tasks. These beliefs are supported by emerging research exploring the educational potential of generative AI in medical education [48,49]. Overall, while the current use remains focused on enhancement, there is growing confidence that AI integration could progress toward more transformative applications.

### Practical Implications

The findings have several practical implications for medical education policy and practice in the United Arab Emirates. Faculty members’ generally positive attitudes toward AI demonstrate institutional readiness for structured adoption; however, the current use remains primarily task-enhancing rather than transformative. Medical schools should therefore develop comprehensive, curriculum-aligned strategies that support higher-level educational transformation through simulation-based learning, adaptive feedback, and decision-making support [50].

To support this transformation, institutions should prioritize faculty development through targeted training programs, interactive workshops, and interdisciplinary collaboration with AI specialists [51]. These efforts should focus on prompt engineering, ethical and critical evaluation of AI-generated outputs, and the effective integration of AI with learning objectives [52]. Such initiatives will not only enhance pedagogical innovation but also empower faculty to guide students in the responsible and informed use of AI.

At the policy level, medical schools need to establish frameworks addressing academic integrity, data privacy, and equitable access [53]. These policies must be informed by UAE-specific cultural and ethical considerations to ensure that AI adoption remains contextually relevant and socially responsible [54]. Moreover, equity-related concerns must be proactively addressed by improving digital literacy and ensuring fair access to AI tools among all students [44].

Fostering interdisciplinary collaboration among educators, AI specialists, and policymakers will be essential to ensure that the integration of AI in medical education evolves in a balanced, ethical, and inclusive manner, enhancing both learning quality and institutional sustainability [55].

### Limitations and Future Research Recommendations

While this study provides novel insights into faculty perceptions of AI integration in medical education, several limitations should be noted. Although our study included faculty from both public and private institutions, providing some diversity in institutional perspectives, the sample was limited to 2 universities in the United Arab Emirates. Future research should include a broader representation of medical schools across the region to enhance transferability. Second, the study relied on qualitative data, offering an in-depth understanding but not providing quantifiable measures of attitudes or behaviors. Third, given the rapidly evolving nature of generative AI technologies, participants’ experiences and perceptions may change as tools advance and institutional policies develop. Continuous monitoring and follow-up research are therefore necessary to examine how faculty adoption evolves over time. The findings should be interpreted within the context of qualitative inquiry, where the goal is analytic depth and contextual understanding rather than statistical generalizability. Although the sample was limited to 10 faculty members from 2 institutions, the study aimed to generate rich, context-specific insight rather than represent the full UAE medical faculty landscape. Readers should therefore consider the findings in terms of their transferability to similar educational settings rather than population-level generalization.

Future studies could adopt multi-institutional or cross-national designs to capture a broader range of perspectives and contextual influences. Additionally, exploring how AI integration affects learner outcomes, including motivation, critical thinking, and professionalism, would provide a more comprehensive understanding of its educational impact.

### Conclusion

This study reveals positive views among UAE medical faculty members toward incorporating AI into the curriculum, highlighting its potential to enhance assessment, feedback, planning, and personalized learning. Faculty recognize AI’s growing role for efficiency and educational alignment in preclinical and clinical settings. Early signs of deeper integration, such as adaptive simulations, indicate a shift toward more advanced use. However, concerns about ethics, regulation, culture, and digital equity highlight the need for cautious, context-aware adoption. Future efforts should focus on enhancing institutional readiness and developing inclusive frameworks that foster responsible innovation. These insights can inform policy discussions and practice within similar institutional contexts in the United Arab Emirates and provide a foundation for further research.

## Supplementary material

10.2196/90192 Multimedia Appendix 1 Interview guide.

## Abbreviations

COREQ: Consolidated Criteria for Reporting Qualitative Research
FACETS: Form, AI Use Case, Context, Education Form, Technology, and SAMR
LLMs: large language models
MCQs: multiple-choice questions
NLP: natural language processing
SAMR: Substitution, Augmentation, Modification, Redefinition

## References

[1] Wiljer D, Hakim Z (2019). Developing an artificial intelligence-enabled health care practice: rewiring health care professions for better care. J Med Imaging Radiat Sci.

[2] Darcy AM, Louie AK, Roberts LW (2016). Machine learning and the profession of medicine. JAMA.

[3] Shortliffe EH, Sepúlveda MJ (2018). Clinical decision support in the era of artificial intelligence. JAMA.

[4] Hosny A, Parmar C, Quackenbush J, Schwartz LH, Aerts HJWL (2018). Artificial intelligence in radiology. Nat Rev Cancer.

[5] Niazi MKK, Parwani AV, Gurcan MN (2019). Digital pathology and artificial intelligence. Lancet Oncol.

[6] Hogarty DT, Su JC, Phan K (2020). Artificial intelligence in dermatology—where we are and the way to the future: a review. Am J Clin Dermatol.

[7] Ting DSW, Pasquale LR, Peng L (2019). Artificial intelligence and deep learning in ophthalmology. Br J Ophthalmol.

[8] Leivaditis V, Beltsios E, Papatriantafyllou A (2025). Artificial intelligence in cardiac surgery: transforming outcomes and shaping the future. Clin Pract.

[9] Guo W, Chen Y (2025). Investigating whether AI will replace human physicians and understanding the interplay of the source of consultation, health-related stigma, and explanations of diagnoses on patients’ evaluations of medical consultations: randomized factorial experiment. J Med Internet Res.

[10] Montanaro B, Croce A, Ughetto E (2024). Venture capital investments in artificial intelligence. J Evol Econ.

[11] Omar G, Othman ZK, Kakarash ZA (2024). The transformative impact of artificial intelligence (AI) on enhancing healthcare systems in the Middle East. Acad J Int Univ Erbil.

[12] Alowais SA, Alghamdi SS, Alsuhebany N (2023). Revolutionizing healthcare: the role of artificial intelligence in clinical practice. BMC Med Educ.

[13] Çalışkan SA, Demir K, Karaca O (2022). Artificial intelligence in medical education curriculum: an e-Delphi study for competencies. PLoS One.

[14] Abdellatif H, Al Mushaiqri M, Albalushi H, Al-Zaabi AA, Roychoudhury S, Das S (2022). Teaching, learning and assessing anatomy with artificial intelligence: the road to a better future. Int J Environ Res Public Health.

[15] Valikodath NG, Cole E, Ting DSW, American Academy of Ophthalmology Task Force on Artificial Intelligence (2021). Impact of artificial intelligence on medical education in ophthalmology. Transl Vis Sci Technol.

[16] Perrin J, Petronic-Rosic V (2024). The potential role and restrictions of artificial intelligence in medical school dermatology education. Clin Dermatol.

[17] Leon S, Lee S, Perez JE, Hashimoto DA (2025). Artificial intelligence and the education of future surgeons. Am J Surg.

[18] Crotty E, Singh A, Neligan N, Chamunyonga C, Edwards C (2024). Artificial intelligence in medical imaging education: recommendations for undergraduate curriculum development. Radiography (Lond).

[19] Jowsey T, Stokes-Parish J, Singleton R, Todorovic M (2023). Medical education empowered by generative artificial intelligence large language models. Trends Mol Med.

[20] Liu M, Okuhara T, Chang X (2024). Performance of ChatGPT across different versions in medical licensing examinations worldwide: systematic review and meta-analysis. J Med Internet Res.

[21] Kaya M, Sonmez E, Halici A, Yildirim H, Coskun A (2025). Comparison of AI-generated and clinician-designed multiple-choice questions in emergency medicine exam: a psychometric analysis. BMC Med Educ.

[22] Ebihara H, Kasai H, Shimizu I (2025). Development of a clinical clerkship mentor using generative AI and evaluation of its effectiveness in a medical student trial compared to student mentors: 2-part comparative study. JMIR Med Educ.

[23] McLaughlin JE, Ponte CD, Lyons K (2025). Student perceptions of GenAI as a virtual tutor to support collaborative research training for health professionals. BMC Med Educ.

[24] Li L, Zhang W, Zhang K (2025). The role of generative AI tools in case-based learning and teaching evaluation of medical biochemistry. BMC Med Educ.

[25] Fatima SS, Sheikh NA, Osama A (2024). Authentic assessment in medical education: exploring AI integration and student-as-partners collaboration. Postgrad Med J.

[26] Jackson P, Ponath Sukumaran G, Babu C (2024). Artificial intelligence in medical education—perception among medical students. BMC Med Educ.

[27] Sami A, Tanveer F, Sajwani K (2025). Medical students’ attitudes toward AI in education: perception, effectiveness, and its credibility. BMC Med Educ.

[28] Ahmad MN, Abdallah SA, Abbasi SA, Abdallah AM (2023). Student perspectives on the integration of artificial intelligence into healthcare services. Digit Health.

[29] McKell D, McCoy L, Niño DF (2024). IAMSE artificial intelligence meeting survey: AI’s impact on medical education faculty. Med Sci Educ.

[30] Al Zahrani EM, Elsafi SH, Al Musallam LD (2025). Faculty perspectives on artificial intelligence’s adoption in the health sciences education: a multicentre survey. Front Med (Lausanne).

[31] Brooks JV, Hughes D (2024). Flipping the expert: faculty educator sensemaking during transition to an active learning-based curriculum. BMC Med Educ.

[32] Acosta-Enriquez BG, Ramos Farroñan EV, Villena Zapata LI (2024). Acceptance of artificial intelligence in university contexts: a conceptual analysis based on UTAUT2 theory. Heliyon.

[33] Tong A, Sainsbury P, Craig J (2007). Consolidated Criteria for Reporting Qualitative Research (COREQ): a 32-item checklist for interviews and focus groups. Int J Qual Health Care.

[34] Braun V, Clarke V (2023). Toward good practice in thematic analysis: avoiding common problems and be(com)ing a knowing researcher. Int J Transgend Health.

[35] Gordon M, Daniel M, Ajiboye A (2024). A scoping review of artificial intelligence in medical education: BEME Guide No. 84. Med Teach.

[36] Hamilton ER, Rosenberg JM, Akcaoglu M (2016). The Substitution Augmentation Modification Redefinition (SAMR) model: a critical review and suggestions for its use. TechTrends.

[37] Kung TH, Cheatham M, Medenilla A (2023). Performance of ChatGPT on USMLE: potential for AI-assisted medical education using large language models. PLOS Digit Health.

[38] Knopp MI, Warm EJ, Weber D (2023). AI-enabled medical education: threads of change, promising futures, and risky realities across four potential future worlds. JMIR Med Educ.

[39] Lyo S, Mohan S, Hassankhani A, Noor A, Dako F, Cook T (2025). From revisions to insights: converting radiology report revisions into actionable educational feedback using generative AI models. J Imaging Inform Med.

[40] Parente DJ (2024). Generative artificial intelligence and large language models in primary care medical education. Fam Med.

[41] Chen Y, Esmaeilzadeh P (2024). Generative AI in medical practice: in-depth exploration of privacy and security challenges. J Med Internet Res.

[42] Omar M, Soffer S, Agbareia R (2025). Sociodemographic biases in medical decision making by large language models. Nat Med.

[43] Cau R, Pisu F, Suri JS, Saba L (2025). Addressing hidden risks: systematic review of artificial intelligence biases across racial and ethnic groups in cardiovascular diseases. Eur J Radiol.

[44] Hadar Shoval D (2025). Artificial intelligence in higher education: bridging or widening the gap for diverse student populations?. Educ Sci.

[45] Ahmad SF, Han H, Alam MM (2023). Impact of artificial intelligence on human loss in decision making, laziness and safety in education. Humanit Soc Sci Commun.

[46] Rincón EHH, Jimenez D, Aguilar LAC, Flórez JMP, Tapia ÁER, Peñuela CLJ (2025). Mapping the use of artificial intelligence in medical education: a scoping review. BMC Med Educ.

[47] Ngo TTA, Vo TTA, Phan MT (2025). The psychology of AI adoption in education: university students’ intentions to use large language models for learning from a TAM and TPB perspective. Acta Psychol (Amst).

[48] Holderried F, Stegemann-Philipps C, Herschbach L (2024). A generative pretrained transformer (GPT)-powered chatbot as a simulated patient to practice history taking: prospective, mixed methods study. JMIR Med Educ.

[49] Sardesai N, Russo P, Martin J, Sardesai A (2024). Utilizing generative conversational artificial intelligence to create simulated patient encounters: a pilot study for anaesthesia training. Postgrad Med J.

[50] Tolentino R, Baradaran A, Gore G, Pluye P, Abbasgholizadeh-Rahimi S (2024). Curriculum frameworks and educational programs in AI for medical students, residents, and practicing physicians: scoping review. JMIR Med Educ.

[51] Mir MM, Mir GM, Raina NT (2023). Application of artificial intelligence in medical education: current scenario and future perspectives. J Adv Med Educ Prof.

[52] Chadha N, Popil E, Gregory J, Armstrong-Davies L, Justin G (2025). How do we teach generative artificial intelligence to medical educators? Pilot of a faculty development workshop using ChatGPT. Med Teach.

[53] Franco D’Souza R, Mathew M, Mishra V, Surapaneni KM (2024). Twelve tips for addressing ethical concerns in the implementation of artificial intelligence in medical education. Med Educ Online.

[54] Mondal H (2025). Ethical engagement with artificial intelligence in medical education. Adv Physiol Educ.

[55] Ahsan Z (2025). Integrating artificial intelligence into medical education: a narrative systematic review of current applications, challenges, and future directions. BMC Med Educ.

