# Supplementary material for: AI Integration in Undergraduate Medical Education: Qualitative Study of Faculty Perspectives in the United Arab Emirates
Source: JMIR Med Educ. 2026 Aug 13;12:e90192. doi: 10.2196/90192 (PMC13472525; doi:10.2196/90192)
Supplement: Supplementary file 1 [file mededu-v12-e90192-s001.docx]

**Category 1: Enhancing Medical Education Quality**

1. How can AI enhance the quality of medical education?
2. how should its value be communicated to students?
3. How can AI help students develop critical skills such as decision-making, problem-solving, and clinical reasoning?
4. What opportunities does AI offer for personalized learning in medical education?
5. How can AI assist students in staying updated with rapidly changing medical knowledge?

**Category 2: Curriculum Design and Delivery**

5. What specific learning objectives should be outlined for an AI-integrated medical curriculum?
6. How should AI concepts and applications be integrated into the existing medical curriculum?
7. What balance should be struck between theoretical knowledge of AI and its practical applications?
8. What role should interdisciplinary collaboration (e.g., with computer science) play in the AI curriculum?

**Category 3: Teaching and Resources**

9. Who should be responsible for teaching AI to medical students, and what qualifications should they have?
10. What resources and infrastructure are necessary for effectively delivering an AI-integrated curriculum?
11. How can medical simulations and AI-based tools enhance experiential learning for students?
12. What strategies can be used to train faculty to teach AI effectively, especially if they lack prior experience?

**Category 4: Evaluation and Assessment**

13. How should the effectiveness of an AI curriculum be assessed?
14. What metrics or indicators should determine the success of AI integration in medical education?
15. How can assessments ensure students understand AI's practical and ethical implications?

**Category 5: Ethical and Practical Considerations**

16. How should ethical considerations in AI, such as bias and patient safety, be taught to students?
17. What role can AI play in preparing students to address disparities in healthcare access?
18. How should students be trained to critically evaluate AI tools used in clinical practice?

**Category 6: Future Preparedness and Relevance**

19. How does AI integration prepare students for future challenges in healthcare, such as precision medicine and big data?
20. How can medical schools ensure the AI curriculum remains relevant as technology evolves?
